# On the morphological relations of the Achilles tendon and plantar fascia via the calcaneus: a cadaveric study

**DOI:** 10.1038/s41598-021-85251-0

**Published:** 2021-03-16

**Authors:** A. Singh, J. Zwirner, F. Templer, D. Kieser, S. Klima, N. Hammer

**Affiliations:** 1 Department of Anatomy, University of Otago, Dunedin, New Zealand; 2 Department of Orthopedic Surgery and MSM, Christchurch Hospital, Christchurch, New Zealand; 3 Orthopaedicus, Leipzig, Germany; 4 Department of Orthopedic and Trauma Surgery, University of Leipzig, Leipzig, Germany; 5 Department of Clinical and Macroscopic Anatomy, Medical University of Graz, Harrachgasse 21, 8010 Graz, Austria; 6 Fraunhofer Institute for Machine Tools and Forming Technology, Section of Medical Engineering, Dresden, Germany; 7 Institute of Legal Medicine, University Medical Center Hamburg Eppendorf, Hamburg, Germany

**Keywords:** Anatomy, Musculoskeletal system, Bone, Cartilage, Ligaments

## Abstract

Current treatments of plantar fasciitis are based on the premise that the Achilles tendon (AT) and plantar fascia (PF) are mechanically directly linked, which is an area of debate. The aim of this study was to assess the morphological relationship between the AT and PF. Nineteen cadaveric feet were x-ray imaged, serially sectioned and plastinated for digital image analyses. Measurements of the AT and PF thicknesses and cross-sectional areas (CSA) were performed at their calcaneal insertion. The fiber continuity was histologically assessed in representative subsamples. Strong correlations exist between the CSA of the AT and PF at calcaneal insertion and the CSA of PF’s insertional length (r = 0.80), and between the CSAs of AT’s and PF’s insertional lengths. Further correlations were observed between AT and PF thicknesses (r = 0.62). This close morphological relationship could, however, not be confirmed through x-ray nor complete fiber continuity in histology. This study provides evidence for a morphometric relationship between the AT and PF, which suggests the presence of a functional relationship between these two structures following the biological key idea that the structure determines the function. The observed morphological correlations substantiate the existing mechanical link between the AT and PF via the posterior calcaneus and might explain why calf stretches are a successful treatment option for plantar heel pain.

## Introduction

The Achilles tendon (AT) and plantar fascia (PF) play a vital role in the load distribution of the foot; both the AT and PF are important in absorbing mechanical shock, as well as stabilizing and preventing the collapse of the longitudinal arch during propulsion^1^. The PF forms a primary structure supporting the medial longitudinal arch of the foot, along with intrinsic muscles of the foot^2,3^. The PF reduces the effect of ground reaction force on the metatarsal heads^4,5^. The AT also has the ability to store and release elastic potential energy during movement, which aids in metabolic energy saving^4^. However, there is debate regarding the morphological and functional relationship between the AT and PF and whether these two structures are connected.

A number of studies have found a biomechanical link between the AT and PF by measuring the load-deformation properties in the PF under various conditions of the AT^5–8^. This biomechanical link provides grounds for hypothesizing that there is continuity between the AT and PF^9^. Indeed, some authors have described a lifelong continuity between the two structures^10–12^ whilst others only agree of the continuity being in a fetal or neonatal population^13,14^, and others concluding that the connection ceases by late adulthood^15^. However, none of the previous studies systematically compared the thicknesses and cross sections of the AT and the PF in the area of their respective calcaneal insertions to assess whether these are linked. Previously, the connection between the AT and PF has been described both as a direct tissue link^10–15^, as well as a trabecular meshwork^16^. The former involves connection via connective tissue whereas the latter details patterns within the trabeculae of the calcaneus, which are aligned in the direction of the fascicles from the AT to the PF. Moreover, there is conflicting evidence of correlations existing between traits such as age, sex and body size against the thicknesses and CSAs of the AT and PF^17–22^. Conclusively, if there are significant correlations found between the thicknesses or CSAs of the AT and PF, it will provide additional evidence on a morphological relationship between the two structures, furthermore implying a functional one according to the concept of ‘structure and function’.

Moreover, the distinct morphology of both the AT and PF in the context of foot kinematics appears to predispose the onset of heel pain and plantar fasciitis^15^. Pain relief in plantar fasciitis often involves conservative treatment, including stretching exercise^3,23^. These treatments are already based on the premise that the AT and PF are mechanically linked; thus, it is important to demonstrate the relationship between the AT and the PF. Furthermore, if an anatomical connection is found, it may inform surgical treatments, e.g., for surgical treatment of insertional Achilles tendinopathy^13,14^.

The aim of this cadaveric study was to substantiate the morphological relationship between the AT and PF, combining techniques of clinical imaging with dissection and histology. Morphometric measurements were taken of the AT and PF both at their point of insertion in the calcaneus and 10 mm proximal (AT) and distal (PF) of it. The insertion areas of the AT and PF were histologically processed to assess for a continuity of the collagen fibers of these two structures and x-ray imaging was used to see the presence of the aforementioned osseous link. We hypothesized that the AT and PF may act as a morphological and thus functional complex via a continuous connection, which may be shown from the AT collagen fiber bundles via the trabecular bone arrangement of the calcaneus to the PF fiber bundles, based on cross section measurements of these structures. A greater understanding on the functionality of this region will contribute to successful treatment of pathologies such as plantar fasciitis.

## Methods

### X-ray imaging, computed tomography and assessment criteria

**Figure 1:**
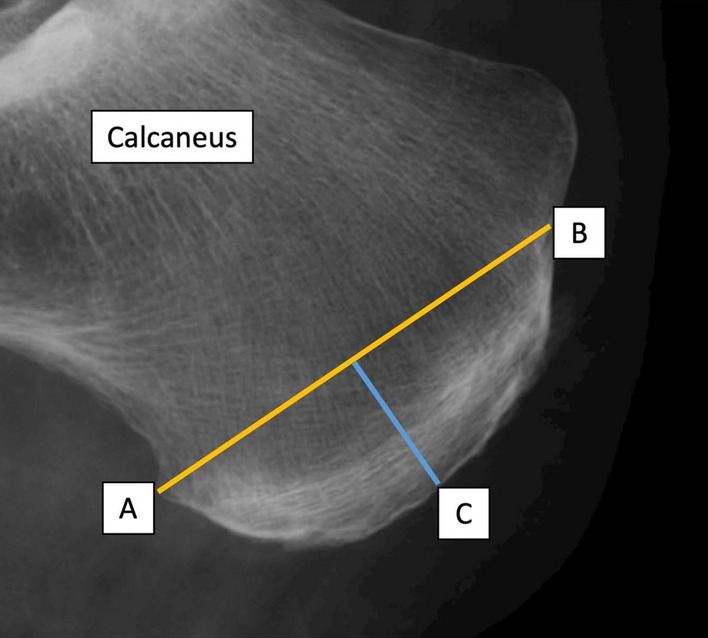
A representative x-ray of the right calcaneus of one specimen is depicted. The midpoint of a line (yellow) drawn from the insertion of the PF into the calcaneus (A) to the insertion of the AT into the calcaneus (B) is where a perpendicular line (blue) was drawn to measure the thickness (C) of the apophysis of the calcaneus.

19 feet from 16 cadavers (9 males, 7 females; age at death 79 ± 14, age range 38 to 94 years) were used; both feet were taken from three cadavers, the remaining were retrieved unilaterally. All cadavers in this study were of Caucasian descent. Ethical approval was granted by the University of Otago Human Ethics Committee (Health) (ref: H17/20) prior to specimen collection and in accordance with national/international/institutional guidelines or the Declaration of Helsinki in the manuscript. Written and informed consent was obtained from the body donors, while being alive, for their post mortem donation for teaching and research purposes in conjunction with the New Zealand Human Tissue Act (2008). Māori consultation for the project was sought from the Ngāi Tahu Research Consultation Committee. The feet were embalmed in a fixative composed of ethanol, phenoxyethanol and formaldehyde were first imaged using a digital lateral plain CARESTREAM DRX-1C x-ray system (Carestream Health, Rochester, NY, USA; spatial resolution: 98 μm; FOV: 130 × 160 mm; tube voltage: 57 kV; Q: 16 mAs^24^). The digital lateral plain film x-rays were analyzed using OsiriX (Pixmeo Sarl, Geneva, Switzerland). The thickness of the apophysis of the calcaneus was measured by first creating a line between the most proximal and distal insertional points of the AT and PF, respectively. A perpendicular line was then created from the midpoint of this line to the edge of the calcaneus, to systematically measure the thickness of the radio-dense trabeculae orientated from posterior to inferior. This method of measurement was chosen to standardize measurements as the calcaneal apophysis has a structurally inhomogeneous anatomy with differing radii. See Fig. 1.

Six calcanei were retrieved from six cadavers aged 76 to 83 years (3 females, 3 males) and micro-computed tomography-scanned (micro-CT-scanned) using a SkyScan 1276 system (Bruker, Billerica, MA, USA; voxel resolution: 36 μm; tube voltage: 85 kV; tube current: 200 μA; slice thickness: 36 μm; rotation: 360°; filter: 1 mm-Al; rotation step: 0.5; exposure time: 223 ms; average scanning time 1.1 h^24^). Following this, the trabecular orientation of the superficial posterior-inferior calcaneus was described in a sagittal, transverse and coronal plane to assess the spatial orientation of the trabeculae.

### Preparation of plastination samples and assessment criteria

Of these 19 feet, one was excluded from being serially sectioned as it had a metal implant resulting from fibular fracture treatment. The remaining 18 feet were embedded in 25% (by weight) gelatin mixture after being precooled at 4 °C, and being frozen at − 20 °C, − 80 °C, respectively over five days. Following this, the feet were serially sectioned in the sagittal plane using a band saw (Butcher Boy B16, Butcher Boy Machines International LLC, Selmer, TN, USA). The specimens were continuously cooled during the cutting by immersion in liquid nitrogen to prevent the formation of artefacts in the serial sections. The thickness of each of the slices averaged 2.50 mm. Between consecutive slices, the amount of tissues removed by the saw band was 0.74 mm (slice interval). The tibia and fibula were aligned, and the foot was kept in a neutral position to the end that the blocks were sectioned in a parallel manner. In the next step, the sections were photographed for digital analysis (Canon EOS 7D equipped with a Canon EF-S 18–55 mm F/4–5.6 IS STM Lens, Ōta, Tokyo, Japan).

The images of the plastinated sections were analyzed using Measure (Datinf, Tübingen, Germany) on a Wacom tablet (Wacom Co., Ltd., Kazo, Saitama, Japan), as described previously^25^. This focused on the measurements of thicknesses, distances and cross-sectional areas (CSA; defined as the areas perpendicular to the fiber orientation) of the AT and PF at different points.

**Figure 2:**
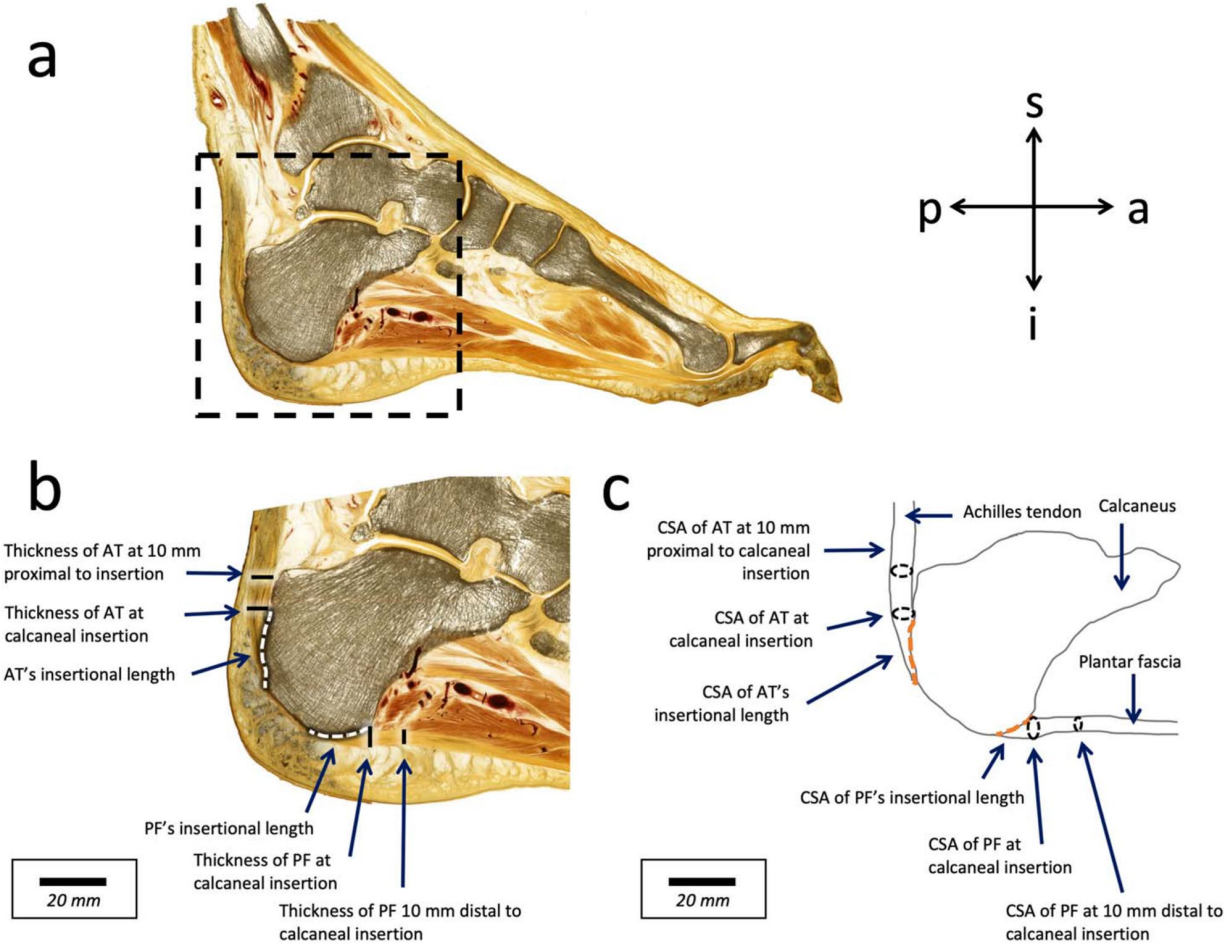
(**a**) Representative section of an E12-plastinate in the sagittal plane. Image (**b**) displaying the calcaneus and the surrounding tissues, is a magnification of the dotted box shown in the image (**a**). The following six measurements used in this study are labelled: Thickness of Achilles tendon (AT) at 10 mm proximal to calcaneal insertion, the thickness of AT at calcaneal insertion, AT’s insertional length, plantar fascia’s (PF) insertional length, the thickness of PF at calcaneal insertion, and the thickness of PF at 10 mm distal to calcaneal insertion. The white arrows highlight the aligned dense trabeculae in the superficial posterior-inferior calcaneus. Image (**c**) is a schematic diagram of the calcaneus, with complementary AT and PF cross-sectional areas (CSA) for the distances of the measurements given in a. *a* anterior; *i* inferior, *p* posterior; s, superior.

The following six measurements for both the AT and PF were taken (see Fig. 2):

1. *Thickness of the AT and PF at 10 mm proximal and 10 mm distal to calcaneal insertion,* respectively: thicknesses of the AT and PF in all sections were measured perpendicular to the collagen fiber arrangement at 10 mm proximal to the AT insertion and 10 mm distal to the PF insertion, respectively; 10 mm was chosen as it excluded any obliquity of the two structures relating to their insertion. The thicknesses at these points were then averaged to produce the reported thickness of the AT and PF at 10 mm proximal and distal to calcaneal insertion.
2. *Thickness of the AT and PF at calcaneal insertion*: thicknesses of the AT and PF in all sections were measured perpendicular to the collagen fiber arrangement at the most proximal and distal areas of the calcaneal insertion of the AT and PF, respectively. The thicknesses at these points were then averaged to produce the reported thickness of the AT and PF at calcaneal insertion.
3. *AT and PF’s insertional length*: AT and PF length at insertion, spanning the distance between the most proximal and the most distal insertion of the calcaneus, respectively. These distances were measured in all consecutive sections where these individual structures were found and then averaged for this distance measurement.
4. *CSA of the AT and PF at 10 mm proximal and 10 mm distal to calcaneal insertion, *respectively: the CSAs were calculated from the aforementioned two-dimensional measurements, adding the individual thicknesses of all sections (2.50 mm) and the respective slicing intervals (0.74 mm).
5. *CSA of the AT and PF at insertion*: the CSAs were calculated from the aforementioned two-dimensional measurements, adding the individual thicknesses of all sections (2.50 mm) and the respective slicing intervals (0.74 mm).
6. *CSA of AT and PF’s insertional length*: the CSAs were calculated from the aforementioned two-dimensional measurements, adding the individual thicknesses of all sections (2.50 mm) and the respective slicing intervals (0.74 mm).

Finally, all of the abovementioned measurements were correlated with the thickness of the calcaneal apophysis that was obtained from the X-ray images.

### Preparation of histology samples, dissection and assessment criteria

Sections were removed from each of the specimens to assess for the presence of continuity of collagen fiber orientation reaching from the AT via the calcaneus into the PF. To this end, sections from 12 feet were retrieved from the AT-calcaneus-PF complex, dehydrated and paraffin wax embedded for serial sectioning at 10 μm. These specimens were then stained using Giemsa and silver staining according to Romeis^26^. Images of these sections were analyzed applying a semiquantitative approach, assessing for visible continuity of the fiber alignment using a three-stage classification system: discontinuous, partly continuous, and fully continuous. Furthermore, representative sections of each of the specimens were further processed in E12 resin using an epoxy sheet plastination technique described elsewhere^27^. The left AT, calcaneus and PF of an 84-year-old male were exposed. Subsequently, the calcaneus was carefully removed to assess, whether there is a soft tissue connection between the AT and the PF.

### Data analyses

Data analysis was conducted using Prism version 8 (GraphPad Software Inc., La Jolla, CA, USA) and Microsoft Excel version 16.24 (Microsoft Corporation, Redmond, WA, USA). Statistical tests applied for correlations were one-tailed Spearman’s rho and one-tailed Pearson’s correlation for non-normally distributed and normally distributed data, respectively. These tests were used to assess for correlations between average thicknesses, distances and CSA values of AT at insertion, AT at 10 mm proximal to the calcaneal insertion, AT’s insertional length, PF at 10 mm distal to the calcaneal insertion, PF at insertion, and PF’s insertional length*.* The measurement of the apophyseal (cancellous bone) thickness from the digital x-rays was furthermore correlated. The assessment of continuity of the fiber alignment in the AT-calcaneus-PF complex was assessed using Cramer’s V. *P* values of ≤ 0.05 were considered statistically significant. The correlation coefficients were classified as follows 0.00–0.10: negligible correlation, 0.10–0.39: weak correlation, 0.40–0.69: moderate correlation, 0.70–0.89: strong correlation, 0.90–1.00: very strong correlation^28^.

## Results

Measurements were obtained from all 18 specimens, with a total of 24 to 37 sections per foot. Within these serial sections, insertions of the AT and PF were found at varying extent, indicating differences in the individual dimensions of both structures over their medio-lateral course. A number of significant correlations were found in statistical testing.

### Cross-section measurements of the Achilles tendon and plantar fascia correlate closely

**Figure 3:**
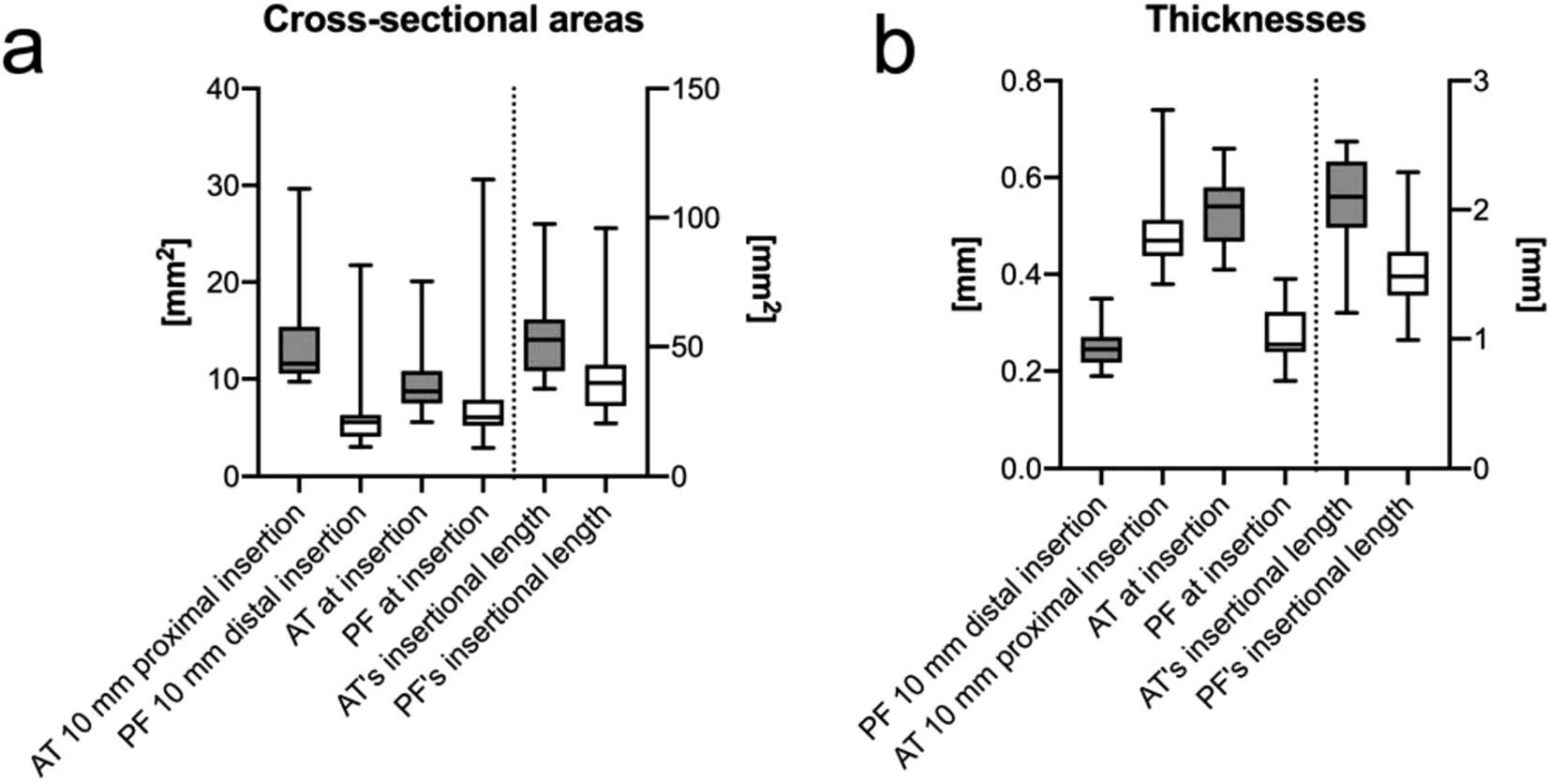
The cross-sectional areas (**a**) and thicknesses (**b**) of the samples are graphically depicted. *AT* Achilles tendon; *PF* plantar fascia.

**Figure 4:**
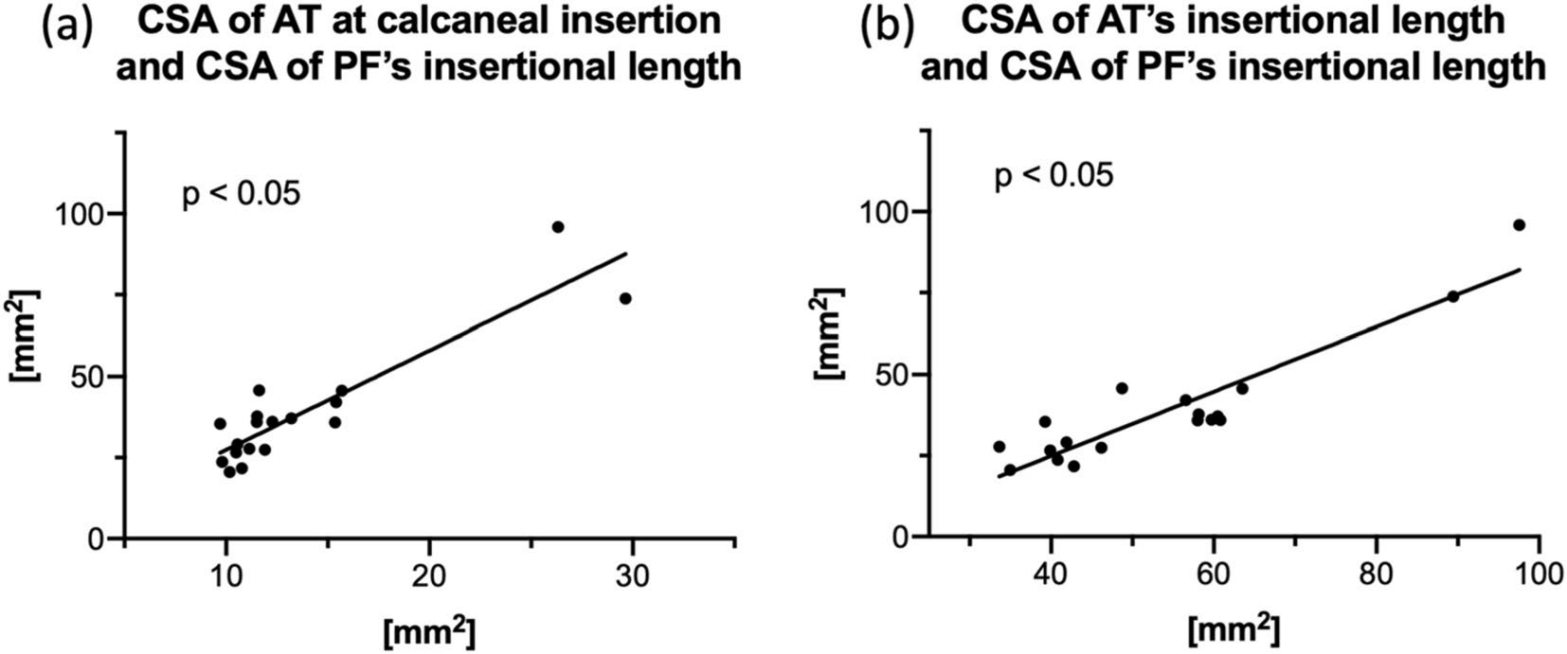
Graph (**a**) shows the cross-sectional area (CSA) of the Achilles tendon (AT) at calcaneal insertion in relation to the CSA of plantar fascia’s (PF) insertional length (r = 0.80, p ≤ 0.001). Graph (**b**) shows the CSA of AT’s insertional length in relation to CSA of PF’s insertional length (r = 0.84, p ≤ 0.001).

Of the given CSA measurements, most values correlated moderately to strongly and highly significantly (p < 0.001), with correlation coefficients r ≥ 0.51 for all comparisons which were statistically significant. Detailed statistically significant correlations are given in Table 1 and the absolute measures graphically depicted in Fig. 3. Of interest, strong correlations were observed between the AT and PF, particularly between the CSA of AT at calcaneal insertion and the CSA of PF’s insertional length (r = 0.80), and between the CSA of AT’s insertional length and the CSA of PF’s insertional length (r = 0.80). These correlations are depicted as graphs in Fig. 4.

**Table 1:** Statistically significant (p ≤ 0.05) correlations between cross-sectional area (CSA) measurements of Achilles tendon (AT) and plantar fascia (PF); Fig. 2 provides a graphical depiction of the measurement sites.

Correlated parameters	r	*p*
CSA of AT at 10 mm proximal to calcaneal insertion vs. CSA of AT at calcaneal insertion	0.68	***
CSA of AT at 10 mm proximal to calcaneal insertion vs. CSA of AT’s insertional length	0.72	***
CSA of AT at calcaneal insertion vs. CSA of AT’s insertional length	0.84	***
CSA of AT at 10 mm proximal to calcaneal insertion vs. CSA of PF at calcaneal insertion	0.33	n/s
CSA of AT at 10 mm proximal to calcaneal insertion vs. CSA of PF at 10 mm distal to calcaneal insertion	0.51	*
CSA of AT at 10 mm proximal to calcaneal insertion vs. CSA of PF’s insertional length	0.76	***
CSA of AT at calcaneal insertion vs. CSA of PF at 10 mm distal to calcaneal insertion	0.60	***
CSA of AT at calcaneal insertion vs. CSA of PF at calcaneal insertion	0.61	***
CSA of AT at calcaneal insertion vs. CSA of PF’s insertional length	0.80	***
CSA of AT’s insertional length vs CSA of PF at 10 mm distal to calcaneal insertion	0.56	***
CSA of AT’s insertional length vs. CSA of PF at calcaneal insertion	0.51	*
CSA of AT’s insertional length vs. CSA of PF’s insertional length	0.80	***
CSA of PF at 10 mm distal to calcaneal insertion vs. CSA of PF’s insertional length	0.75	***
CSA of PF at calcaneal insertion vs. CSA of PF at 10 mm distal to calcaneal insertion	0.82	***
CSA of PF at calcaneal insertion vs. CSA of PF’s insertional length	0.60	*

*P* p-value; *r* correlation coefficient; *n/s* not significant.

*p ≤ 0.05, **p ≤ 0.01, ***p ≤ 0.001.

### Correlations between Achilles tendon and plantar fascia distance measurements are less distinct and only evident for their respective insertion thicknesses and insertion lengths

Beyond the measurements indicating consistency within the AT and PF (i.e., consistent changes in intrinsic distances), further correlations were found when comparing AT and PF insertion thickness and AT and PF insertion length. These correlations were weaker compared to the measurements of the CSA, indicated by their correlation coefficients. The measurements are summarized in Table 2 and the absolute measures graphically depicted in Fig. 3.

**Table 2:** Statistically significant (p ≤ 0.05) correlations between average Achilles tendon (AT) and plantar fascia (PF) thicknesses.

Correlated parameters	R	*p*
Thickness of AT at 10 mm proximal to calcaneal insertion vs. thickness of AT at calcaneal insertion	0.69	***
Thickness of AT at 10 mm proximal to calcaneal insertion vs. thickness of AT’s insertional length	0.35	n/s
Thickness of AT at calcaneal insertion vs. thickness of AT’s insertional length	0.45	*
Thickness of AT at 10 mm proximal to calcaneal insertion vs thickness of PF at calcaneal insertion	0.34	n/s
Thickness of AT at 10 mm proximal to calcaneal insertion vs. thickness of PF at 10 mm distal to calcaneal insertion	− 0.09	n/s
Thickness of AT at 10 mm proximal to calcaneal insertion vs. thickness of PF’s insertional length	0.07	n/s
Thickness of AT at calcaneal insertion vs. thickness of PF at 10 mm distal to calcaneal insertion	0.20	n/s
Thickness of AT at calcaneal insertion vs. thickness of PF at calcaneal insertion	0.55	**
Thickness of AT at calcaneal insertion vs. thickness of PF’s insertional length	0.36	n/s
Thickness of AT’s insertional length vs thickness of PF at 10 mm distal to calcaneal insertion	0.32	n/s
Thickness of AT’s insertional length vs. thickness of PF at calcaneal insertion	0.01	n/s
Thickness of AT’s insertional length vs. thickness of PF’s insertional length	0.62	**
Thickness of PF at 10 mm distal to calcaneal insertion vs. thickness of PF’s insertional length	0.59	**
Thickness of PF at calcaneal insertion vs. thickness of PF at 10 mm distal to calcaneal insertion	0.54	**
Thickness of PF at calcaneal insertion vs. thickness of PF’s insertional length	0.07	n/s

*P,* p-value; *r,* correlation coefficient; *n/s,* not significant.

*p ≤ 0.05, **p ≤ 0.01, ***p ≤ 0.001.

### Calcaneal apophysis thickness and Achilles tendon insertional length correlate in lateral x-ray

Only one significant correlation was found between the thickness of the calcaneal apophysis and the maximum insertional length of the AT (r = 0.43; p = 0.04). No other variable correlated with the thickness of the calcaneal apophysis. From a morphological perspective, the trabeculae of the calcaneus were observed to be aligned along the direction of the fascicles in the AT towards the PF in all specimens.

### While a soft tissue connection between Achilles tendon and planter fascia is evident, an osseous link via the superficial posterior-inferior calcaneus can be suspected

**Figure 5:**
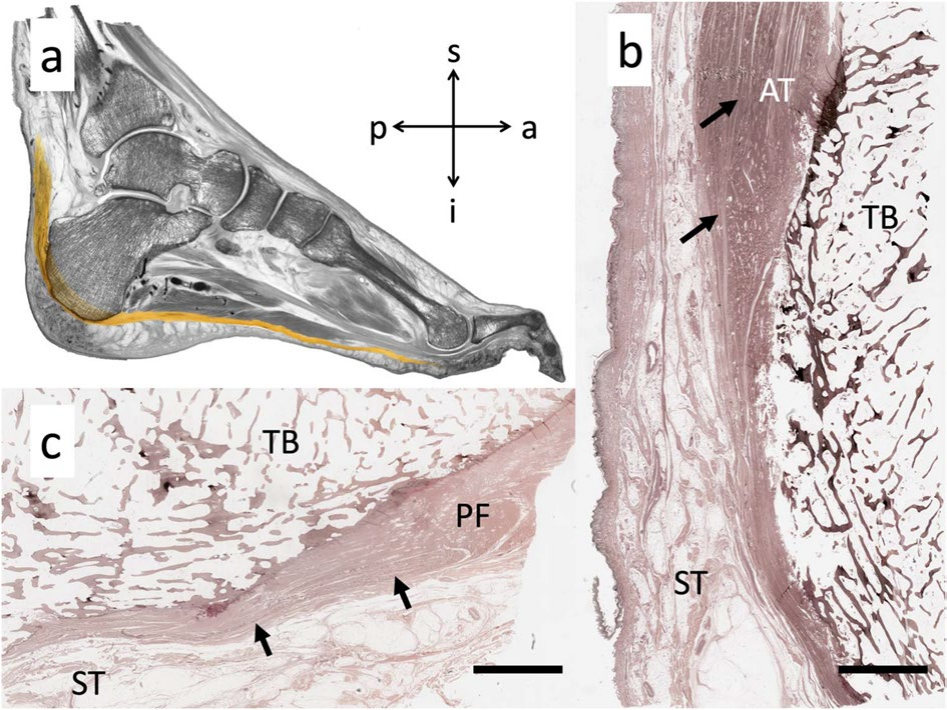
Image (**a**) shows a representative section of an E12-plastinate in the sagittal plane in black and white with the area of interest in this study in natural color. The aligned dense trabeculae within the superficial posterior-inferior calcaneus appear to be the continuation of the Achilles tendon (AT) towards the plantar fascia (PF). The location of the histological sections 4b and 4c is indicated with dotted squares. Image (**b**) shows a silver staining of calcaneal trabecular bone (TB) and the inserting AT. Superficial to the calcaneus, the AT subcutaneous tissue (ST) is depicted. The black arrows point to the collagen bundles of the AT running from superior to inferior in this slice. Image (**c**) shows a silver stained slice of the foot in the area of the PF. Several bundles of collagens jointly run in an anisotropic fashion (black arrows). Scale bar, 5 mm.

**Figure 6:**
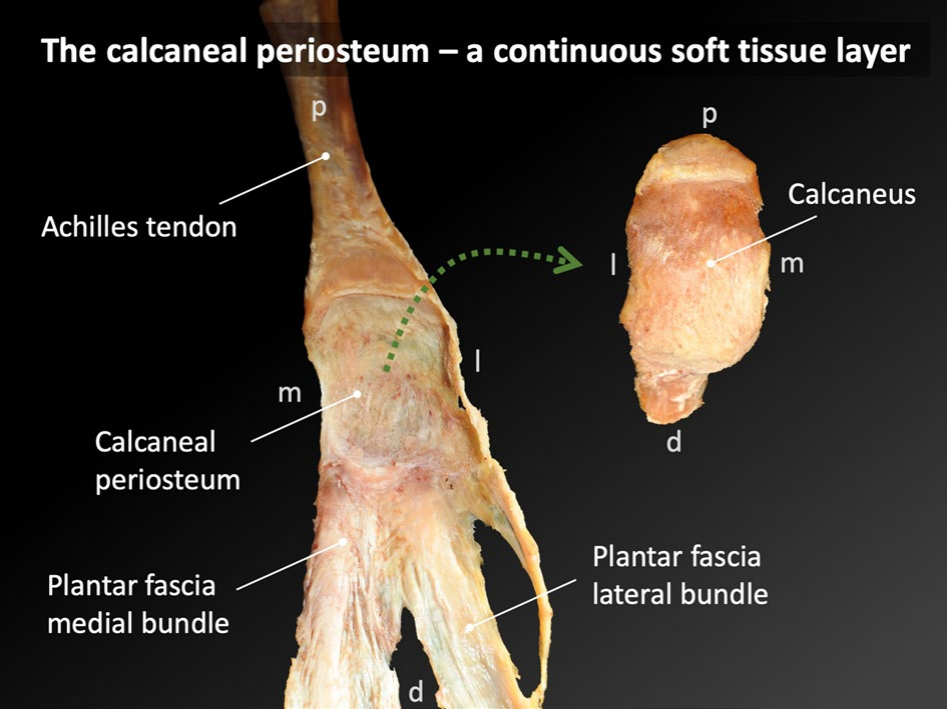
The soft tissue connection between the Achilles tendon (AT) and the plantar fascia (PF) is shown after the calcaneus was removed. The macroscopically most superficial layer of the AT (paratenon of the AT) continues as the calcaneal periosteum to merge with the PF, thereby forming a soft tissue connection between the two. *d* distal; *l* lateral; *m* medial; *p * proximal.

**Figure 7:**
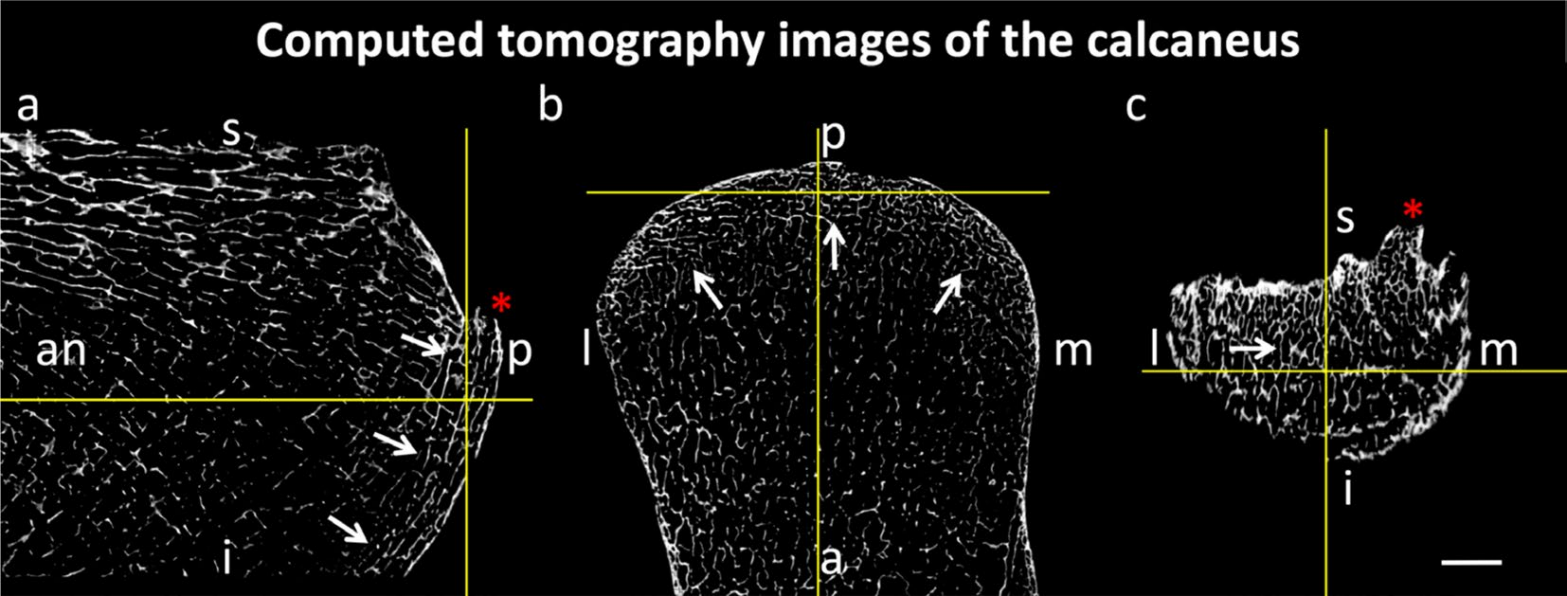
Computed tomography images of the calcaneus are depicted. The aligned dense trabeculae (white arrows) within the superficial posterior calcaneus are shown in a sagittal (**a**), transverse (**b**) and coronal (**c**) plane (white arrows). The center of the yellow cross marks the same spatial point within the three different planes. *an* anterior; i, inferior; *l* lateral; *m* medial; *p* posterior; *s* superior; red asterisk, calcaneal spur; scale bar: 5 mm.

Of all specimens, 73% of the AT and 88% of the PF histological sections were classified as partially continuous with the trabecular arrangement of the inferior calcaneus; the collagen bundles were seen to be directly in line with the trabecular arrangement. Sections from the remaining specimens were classified as discontinuous as all fibers were observed to terminate at the bone. See Fig. 5. The dissection revealed that the paratenon of the AT continues as the calcaneal periosteum to then merge with the PF, which evidences the soft tissue connection between AT and PF. See Fig. 6. All of the investigated micro-CT samples presented aligned dense trabeculae within the superficial posterior-inferior calcaneus. See Fig. 7.

## Discussion

This study provides evidence for a morphometric connection between the AT and PF on a quantitative (statistical) level, hence suggesting the presence of a functional relationship between these two structures. As evidenced statistically, this close morphological coherence is greater than what would be expected on the basis of sex, age or body size. Although evidence of a such relationship through x-ray or complete fiber continuity in histology could not be demonstrated, there was within-structure consistency of the CSA, and also the average and maximum thicknesses of these structures. There were moderate to strong and statistically significant correlations found between the CSA of most points. Some correlations were found between the thicknesses of the AT and PF. Additionally, there was also a statistically significant moderate correlation found between the insertional lengths of the AT and PF at the calcaneus.

As mentioned above, the x-ray images and the histology lacked confirming a relationship between the AT and PF. However, the low resolution of the x-ray images may have impacted the results, with an incomplete depiction of the investigated structures. Moreover, in the histological images, the evidence of complete fiber continuity was possibly not seen as the collagen bundles are unlikely to meet in one single sagittal plane throughout their course from the AT to the PF via the calcaneus. It is likely that these collagen bundles travel along a transverse (mediolateral) axis during their course, especially since the AT has been shown to have a twisted structure^29,30^.

The thickness of the calcaneal apophysis in the x-ray images appeared not to correlate with the thickness nor CSA measurements of the AT and PF. However, based on the plastinated (shown in Fig. 2) and the histological (shown in Fig. 5) sections here, we can confirm what Milz et al. outlined in their study: in the digital x-rays, there is the presence of patterns within the trabeculae of the superficial posterior-inferior calcaneus, which are aligned along the direction of the fascicles in the AT towards the PF^16^. This is thought to allow for force transmission along the PF to maintain the median longitudinal arch of the foot^16,31^. The alignment of the trabeculae is in accordance with Wolff’s law, which states that the internal trabecular bone adapts to external loadings to reflect the principle direction of strain^31–33^. This osseous line of force transmission complements there being a direct tissue link between the AT and PF below the calcaneus^16^.

Advancing on the topic of a direct tissue link, there was some histological evidence of such a relationship between the AT and PF in this given study. The majority of the images showed that the collagen fibers from both the AT and PF partially continued over the surface of the calcaneus. The dissected foot on this study revealed the continuity of (most likely collagenous) fibers covering the superficial posterior-inferior calcaneus, connecting the AT and PF. However, histologic investigation in this study cannot prove the continuity of these two structures through the organic matrix of the calcaneus. When considering the development of the AT enthesis organ in neonates, the PF has been previously stated to be continuous with the distal part of the AT through a thick layer of heavy collagen fibers^10,15^. However, in the existing literature, it is also noted that both these collagenous structures attach to and continue via the perichondrium of the calcaneus, not the skeletal analgen^10^; this continuity is an example of a functional adaptation which allows for stress to be dissipated over a wider area^10,11^. The AT also flares out at the point of insertion as an adaption to resist the effects of insertional angle change and also to secure skeletal anchorage^11^. The myofascial continuity between the AT and PF is also described in detail by Myers; the merging of one enthesis to another increases the stability of the anchorage^11,34^.

A number of studies have investigated the biomechanical link between the AT and PF by measuring the load-deformation properties in the PF under various conditions of the AT and here found positive correlations^5–8^. It is this biomechanical link that forms the basis of surgical treatment of plantar fasciitis, which is based on an underlying yet poorly understood morphology. Several authors have also commented on the continuity of the AT and PF in the adult foot^10,12,16,34,35^. Stecco et al. also found a strong correlation between the thicknesses of the AT and PF paratenon, which was confirmed by MRI^3^. However, two groups of researchers, Snow et al. and Kim and coworkers, stated a contiguous relationship between the AT and PF only in fetal or infant specimens, respectively^13–15^. Snow et al. agree that a mechanical link exists between the two structures, but not a morphological one, as the connecting collagen fibers lose their continuity with age, which they examined grossly and histologically^15^. However, it was a small study conducted on only ten specimens with three being neonatal. Kim et al. only found partial contiguity in longitudinal individual fibers grossly and through MRI but only in younger feet; there was a statistically significant difference in the mean age in the group with contiguity between the two structures and without contiguity^13,14^.

The results of this given study inform on the relationship between the AT and the PF, playing a role in the treatment of conditions such as Achilles tendinitis and plantar fasciitis^15^. Plantar fasciitis is a painful heel syndrome of an unclear cause and occurs when the PF undergoes a form of pathological degeneration and accounts for 1% of all orthopedic visits^3,36^. The treatment of pain in plantar fasciitis includes exercises that involve calf stretching; these exercises are supported by the fact that 80% of plantar fasciitis cases present with AT tightness^37^. Warren and Carlson et al. stated that tightness and excessive stretching of the AT are risk factors of plantar fasciitis^8,38^. The morphological results of this study support the theory that stretching of the AT and the calf musculature can help to relieve the pain in plantar fasciitis. Also, this study revealed novel observations, e.g., that the AT cross section at the calcaneal insertion strongly correlated with the cross section of the insertion (equals the here measured cross section of the insertional length) or that there was a strong correlation between the cross sections of the AT and PF insertions. It would be of interest whether the systematic measurements stated in this study are different between patients suffering from plantar fasciitis and healthy controls to gain further morphological insights into this inflammation state. As little quantitative measures are available of the AT-calcaneus-PF complex to date, the authors decided to not only include the intuitive comparisons between the cross section of the AT and the PF 10 mm proximal and distal to their respective calcaneal insertions but also compare the measures among each other, e.g., the cross section of the AT at the calcaneal insertion and the cross section of the PF’s insertional length. Moderate to strong correlations were also detected in the latter group, which indicates that the thickness of the AT at its insertion can inform the length of the PF’s calcaneal enthesis. Future studies should assess this correlation in plantar fasciitis patients and evaluate whether this morphological observation is of value for clinicians regarding the diagnosis or prognosis of this condition. Besides this, the anatomy of the posterior heel is important for more precise surgical planning^13,14^. Surgical treatment of plantar fasciitis is reserved for patients who do not respond to conservative treatment and refers to PF release (cutting part of the PF). However, this treatment has been shown to lead to negative consequences on the arch of the foot, and other adverse biomechanical changes^5,8,13,14^.

As a result of these findings, a parallel can be drawn between the morphological features of the knee, hand and ankle. The patella forms the largest sesamoid bone of the body, being embedded in the quadriceps tendon, which becomes the patellar ligament^39^. In this study, the calcaneus has been shown to be attached to the AT, which has partially continued on to form the PF through superficial fibers seen histologically. The dissection performed in this given study showed that the superficial fibers of the AT continue as the calcaneal periosteum to merge with the AT. Hence, the calcaneal periosteum forms a soft tissue “bridge” between the AT and the PF. This is the basis of the concept that the posterior-inferior calcaneus may act functionally as a sesamoid bone similar to the patella, where the bones act as a fulcrum in their respective functional anatomical units^40^. Additionally, fat forms part of the AT enthesis organ, which has been described to be comparable to the fat that is associated with the deep infrapatellar bursa, which helps to minimize stress during locomotion^41^.

### Limitations

Several limitations apply to this study including the relatively small sample size of only 19 feet. Having a larger sample size available would potentially have allowed for obtaining more statistically significant results regarding the thickness correlations between the AT and the PF and calcaneal apophysis. Apart from three specimens, only one foot could be used for the given project in 16 cases. Therefore, a side-comparison of the data was not performed to avoid a potential bias by the very limited number of tissues. Due to the limit of resources available for this project tissues of only 12 feet could be included in the histology part of the study. All cadavers in this study were of Caucasian descent. It should be mentioned that the stated findings might differ for other ethnic groups. Post-mortem changes or artefacts as a result of the embalming (e.g., non-uniform tissue shrinkage caused by formaldehyde) and embedding procedures may also have affected the measurements^42,43^. Even though the structures of interest in this study were clearly recognizable when being analyzed in all here stated methods, the authors cannot exclude that methodology-specific limitations such as the resolution of the x-ray images might have unpreventably affected the here stated results. More sophisticated methods such as histological 3D-reconstructions may have enhanced the data quality of the image analysis.

## Conclusion

In conclusion, there are significant correlations between the measurements of the AT and PF at given points in proximity to the calcaneus and distal of it, suggesting that these two structures are morphologically linked. This morphological-functional link provides a rationale for the treatment of plantar pain conditions such as plantar fasciitis with calf stretching exercises. Since the AT and PF are closely coherent, treatment strategies involving the AT may also have implications for the PF. Our results underline the need for further investigation of less invasive techniques elucidating the morphological-functional relation as one continuous structure. Furthermore, future research work should be done in this area to examine the trabecular arrangement in the apophysis of the calcaneus to further explore the osseous link between the AT and the PF.
